# Dysfunctional Vascular Endothelium as a Driver of Atherosclerosis: Emerging Insights Into Pathogenesis and Treatment

**DOI:** 10.3389/fphar.2021.787541

**Published:** 2021-12-22

**Authors:** Steven R. Botts, Jason E. Fish, Kathryn L. Howe

**Affiliations:** ^1^ Toronto General Hospital Research Institute, University Health Network, Toronto, ON, Canada; ^2^ Institute of Medical Science, University of Toronto, Toronto, ON, Canada; ^3^ Temerty Faculty of Medicine, University of Toronto, Toronto, ON, Canada; ^4^ Department of Laboratory Medicine and Pathobiology, University of Toronto, Toronto, ON, Canada; ^5^ Peter Munk Cardiac Centre, University Health Network, Toronto, ON, Canada; ^6^ Division of Vascular Surgery, Department of Surgery, University of Toronto, Toronto, ON, Canada

**Keywords:** vascular endothelium, endothelial dysfunction, atherosclerosis, disturbed flow, endothelial-to-mesenchymal transition, extracellular vesicles, inflammation resolution, targeted therapy

## Abstract

Atherosclerosis, the chronic accumulation of cholesterol-rich plaque within arteries, is associated with a broad spectrum of cardiovascular diseases including myocardial infarction, aortic aneurysm, peripheral vascular disease, and stroke. Atherosclerotic cardiovascular disease remains a leading cause of mortality in high-income countries and recent years have witnessed a notable increase in prevalence within low- and middle-income regions of the world. Considering this prominent and evolving global burden, there is a need to identify the cellular mechanisms that underlie the pathogenesis of atherosclerosis to discover novel therapeutic targets for preventing or mitigating its clinical sequelae. Despite decades of research, we still do not fully understand the complex cell-cell interactions that drive atherosclerosis, but new investigative approaches are rapidly shedding light on these essential mechanisms. The vascular endothelium resides at the interface of systemic circulation and the underlying vessel wall and plays an essential role in governing pathophysiological processes during atherogenesis. In this review, we present emerging evidence that implicates the activated endothelium as a driver of atherosclerosis by directing site-specificity of plaque formation and by promoting plaque development through intracellular processes, which regulate endothelial cell proliferation and turnover, metabolism, permeability, and plasticity. Moreover, we highlight novel mechanisms of intercellular communication by which endothelial cells modulate the activity of key vascular cell populations involved in atherogenesis, and discuss how endothelial cells contribute to resolution biology – a process that is dysregulated in advanced plaques. Finally, we describe important future directions for preclinical atherosclerosis research, including epigenetic and targeted therapies, to limit the progression of atherosclerosis in at-risk or affected patients.

## Introduction: Atherosclerosis and Endothelial Dysfunction

Atherosclerosis is a chronic inflammatory process in which the accumulation of cholesterol-laden plaque restricts blood flow within the arterial vasculature. The occlusion of arteries by luminal encroachment of expanding plaque or emboli from plaque rupture underlies a spectrum of cardiovascular diseases (CVDs) including myocardial infarction, ischemic cardiomyopathy, stroke, and peripheral vascular disease. Although CVD has remained a leading cause of morbidity and mortality in high-income countries (Piepoli et al., 2016), an epidemiological shift has occurred in recent decades (Dai et al., 2020; Libby, 2021), where improvements in vaccination and treatment of infectious diseases have led to a notable increase in CVD prevalence within low- and middle-income nations. The prominent and evolving burden of atherosclerotic CVD has stimulated continued interest in the identification of cellular mechanisms that govern its pathogenesis, which may aid in the discovery of novel biomarkers and therapeutic targets for CVD prevention, detection, and treatment.

Endothelial cells (ECs) comprise the vascular endothelium, the inner lining of all blood vessels, which forms the interface between systemic circulation and underlying tissues. The quiescent or non-proliferating endothelium, once considered to be dormant outside the settings of vascular development or disease, is now understood to play an active role in maintaining vascular homeostasis by receiving and generating diverse biochemical (i.e., autocrine, paracrine, and endocrine) and mechanical signals (Cahill and Redmond, 2016; Ricard et al., 2021). The systemic functions of the endothelium are numerous, and include the provision of oxygen and nutrients to tissues, regulation of vascular tone and permeability, maintenance of hemostasis and coagulation, induction of angiogenesis, and coordination of the inflammatory response (Cahill and Redmond, 2016; Ricard et al., 2021). These essential processes are modulated by rich crosstalk between ECs and other vascular cell populations, including smooth muscle cells (SMCs), monocytes, and macrophages, which contribute to normal vascular function in physiological settings. Likewise, dysregulated communication between ECs and other vascular cell types is associated with vascular dysfunction and pathological remodeling in CVDs such as hypertension, atherosclerosis, and aneurysm (Jaipersad et al., 2014; Méndez-Barbero et al., 2021).

The pathophysiology of atherosclerosis begins with the perturbed endothelium and is mediated by a cascade of intra- and intercellular signaling events that shape the behaviour of cells within the vasculature (da Luz et al., 2018). Vascular ECs facilitate the active transport of low-density lipoprotein (LDL) to the subendothelial space through transcytosis pathways (Mundi et al., 2018), and LDL accumulation initiates a vascular inflammatory response. Early in atherosclerosis, the endothelium transitions from a quiescent to an activated state in response to proatherogenic stimuli, including oxidized LDL (oxLDL), proinflammatory cytokines, and disturbed flow (Cahill and Redmond, 2016; da Luz et al., 2018). In turn, the activated endothelium plays a critical role in the recruitment of inflammatory cells including T lymphocytes, neutrophils, and monocytes to the arterial intima, the first of which induces the adaptive immune response, and the latter of which gives rise to intimal macrophages (da Luz et al., 2018; Libby, 2021). Subsequent lipid engulfment by macrophages produces foam cells, which undergo necrosis and apoptosis to form the lipid core of the progressing atherosclerotic lesion. Vascular SMCs that comprise the medial layer of arteries migrate to the intima, form fibrous tissue through the production of collagen and elastin, and can also differentiate into macrophage-like foam cells in the developing plaque (Bennett et al., 2016). In this proatherogenic environment, communication between the endothelium and other vascular cell populations stimulates the release of proinflammatory signals, which augment local inflammation and contribute to sustained plaque progression. Intimal thickening occurs during plaque development and creates a hypoxic intraplaque environment, which stimulates angiogenesis of the vasa vasorum – adventitial blood vessels that supply larger arteries – and promotes neovascularization into the vascular wall (Jaipersad et al., 2014). Progressive thinning of the fibrous cap results from an inflammation-associated decrease in collagen synthesis and increase in degradation, which in combination with erosion of the endothelium, contributes to plaque rupture, thrombosis, and obstruction of the affected vessel (Libby, 2021). Although beyond the scope of this review, the role of endothelial dysfunction in plaque rupture (Bentzon et al., 2014; White et al., 2016), as well as the contribution to pathogenesis and the therapeutic potential of the vasa vasorum in treating atherosclerosis (Xu et al., 2015; Boyle et al., 2017; Sedding et al., 2018), has been previously discussed in detail. The pathophysiological relevance of the arterial endothelium has been similarly outlined (Cahill and Redmond, 2016; da Luz et al., 2018; Libby, 2021), however, the specific and modifiable intra- and intercellular mechanisms that mediate endothelial dysfunction and atherogenesis remain to be elucidated.

**FIGURE 1:**
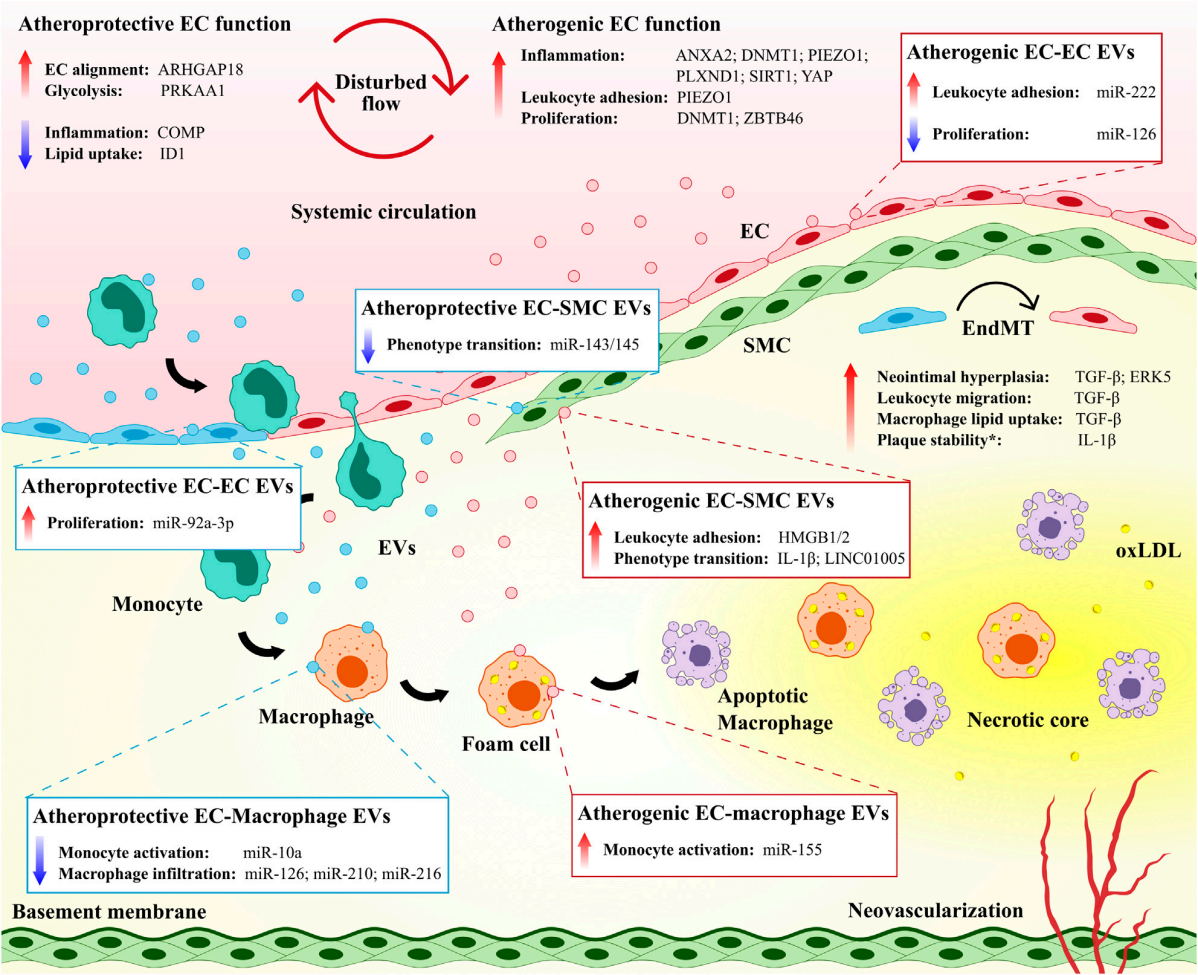
The vascular endothelium directs site-specificity for plaque development and governs plaque progression. Both atheroprotective and atherogenic mechanisms are operative in endothelial cells (ECs) exposed to disturbed flow. EC-derived extracellular vesicles (EVs) mediate atheroprotective and atherogenic intercellular communication among ECs and between ECs and other immune and non-immune cell populations. Endothelial-to-mesenchymal transition (EndMT) contributes to the atherosclerotic disease process but may also maintain plaque stability.* ANXA2, annexin A2; ARHGAP18, Rho GTPase activating protein 18; COMP, cartilage oligomeric matrix protein; DNMT1, DNA methyltransferase 1; ERK5, extracellular signal-regulated kinase 5; HMGB1/2, high mobility group box protein 1/2; ID1, inhibitor of DNA binding 1; IL-1β, interleukin 1 beta; oxLDL, oxidized low-density lipoprotein; PLXND1, plexin D1; PRKAA1, protein kinase AMP-activated catalytic subunit alpha 1; SIRT1, sirtuin 1; SMC, smooth muscle cell; TGF-β, transforming growth factor beta; YAP, yes-associated protein; ZBTB46, zinc finger and BTB domain-containing protein 46.

In this review, we present emerging studies that implicate the vascular endothelium as a driver of atherosclerotic CVD by directing site-specificity of plaque formation and governing plaque progression through intracellular processes. Furthermore, we highlight recent studies that describe intercellular communication between ECs and key vascular cell types involved in atherogenesis (Figure 1), and discuss how ECs participate in resolution biology during plaque development. Finally, we outline outstanding questions and future directions for atherosclerosis treatments, including epigenetic interventions and the targeted delivery of therapeutics to the activated endothelium to resolve vascular inflammation and limit atherosclerotic plaque progression.

## Endothelial Cells Govern Site-Specificity of Atherogenesis

The vascular endothelium is subjected to both tangential (i.e., shear stress) and circumferential forces (i.e., pulsatile stretch) that result from circulating blood flow. ECs at this interface convert mechanical stimuli to biochemical signals through mechanotransduction, which modulates key cellular processes in response to fluctuations in the vascular environment, including proliferation and turnover (Hahn and Schwartz, 2009; Nigro et al., 2011). The development of early atherosclerotic lesions is characteristically localized to regions of the vasculature where laminar blood flow is disturbed (e.g., arterial branch points and lesser curvatures of vessels) (Giddens et al., 1993; VanderLaan et al., 2004), which have previously been associated with decreased expression of endothelial nitric oxide synthase (eNOS) and increased nuclear factor kappa B (NF-κB) activation (Hajra et al., 2000; Collins and Cybulsky, 2001; Won et al., 2007).

### Flow Induces Atherogenic and Atheroprotective Responses in Endothelial Cells

In the setting of undisturbed laminar flow, nitric oxide (NO) is produced by elevated levels of eNOS in ECs and diffuses across cell membranes to modulate the activity of vascular cells, including SMCs and leukocytes (Pan, 2009). NO-mediated activation of soluble guanylate cyclase and S-nitrosylation has been observed to inhibit SMC proliferation (Ignarro et al., 2001), suppress EC inflammation (Matsushita et al., 2003; Kang-Decker et al., 2007), and regulate vascular tone, blood flow, and oxygen delivery to tissues (Stamler et al., 1997; Haldar and Stamler, 2013). Hydrogen sulfide, a gaseous signaling molecule and upstream regulator of NO, is similarly produced under laminar flow conditions (Huang et al., 2015) and has a critical role in reducing vascular inflammation (Zanardo et al., 2006), promoting antioxidative activity (Muzaffar et al., 2008), and limiting the formation of foam cells (Zhao et al., 2011). Laminar flow conditions have also been found to promote EC alignment (Hahn and Schwartz, 2009), and foster a reducing environment that limits oxidative stress (Berk, 2008), tumor necrosis factor α- and signal transducer and activator of transcription 3-induced inflammation (Ni et al., 2003; Yamawaki et al., 2003), and EC turnover via apoptosis (Pi et al., 2004). The transcription factor Kruppel-like factor 2 (KLF2) has emerged as a critical transcriptional mediator of vascular homeostasis that is induced under laminar shear stress and increases eNOS expression and represses adhesion proteins such as vascular cell adhesion molecule 1 (VCAM-1) and E-selectin (SenBanerjee et al., 2004; Berk, 2008), which are induced by NF-κB in regions of disturbed flow (Hajra et al., 2000; Collins and Cybulsky, 2001). In contrast to regions of laminar flow, altered hemodynamics in atheroprone regions has been associated with increased oxidative stress (Förstermann et al., 2017), reorganization of cytoskeletal and cell-cell junction proteins (Chong et al., 2013; Pfenniger et al., 2013), and enhanced senescence and turnover in ECs (Xu, 2009; Warboys et al., 2014). Curiously, hydrogen sulfide has been observed to impair dilation of coronary arteries by reducing NO production under disturbed flow (Chai et al., 2015), although hydrogen sulfide administration has also been shown to inhibit leukocyte adhesion to the endothelium in regions of shear stress by upregulating Akt/eNOS signaling (Go et al., 2012).

Recent studies continue to elucidate a plethora of EC regulatory pathways that induce a proinflammatory and proatherogenic phenotype in response to disturbed flow (Qu et al., 2020; Zhao et al., 2020; Xia et al., 2021) (Figure 1). The mechanosensitive cation channel Piezo1, though essential for coordinating vascular morphogenesis under physiological shear stress in embryogenesis and adulthood (Li J. et al., 2014), has been previously linked to altered flow-induced inflammation and leukocyte recruitment to the endothelium. Indeed, pathological activation of Piezo1 has been associated with induction of downstream integrin α5 and NF-κB pathways, which contributes to endothelial inflammation and the progression of murine atherosclerosis (Albarrán-Juárez et al., 2018), and elevation of intracellular calcium, which results in actin disruption and increased monocyte adhesion in cultured ECs (Swain and Liddle, 2020). Likewise, integrin α5β1-associated phosphorylation of yes-associated protein (YAP) by c-Abl kinase (Li B. et al., 2019), the coupling of integrin α5 to annexin A2 (Zhang C. et al., 2020), and the guidance receptor plexin D1, upstream of EC integrins (Mehta et al., 2020), have been identified as novel mechanisms for endothelial activation under oscillatory shear stress. In contrast, several EC processes confer *in vivo* protection against atherosclerosis under disturbed flow, including inhibition of integrin α5 by cartilage oligomeric matrix protein (Lv et al., 2021), increased glycolysis by protein kinase AMP-activated catalytic subunit alpha 1 (Yang et al., 2018), decreased lipid uptake by the transcription factor inhibitor of DNA binding 1 (Zhang K. et al., 2018), and increased EC alignment by Rho GTPase activating protein 18 (Lay et al., 2019). Many of these preclinical findings hold promise for the development of atherosclerosis treatments through pharmacological modulation of the endothelium (e.g., mitigating atherogenic responses or promoting atheroprotective functions in ECs). However, further investigation is required *in vitro* to determine the generalizability of endothelial responses between vascular cell models (e.g., shared and distinct behaviors of different EC lines under varying flow conditions) (Maurya et al., 2021), as well as *in vivo* to identify translatability in preclinical models and long-term safety and efficacy in individuals with CVD.

### Flow-Mediated Transcriptional and Epigenetic Regulation Modulates Endothelial Proliferation and Turnover

Emerging evidence has also implicated disturbed blood flow in the dysregulation of EC proliferation and turnover during early atherosclerosis. Under physiological conditions, ECs that become senescent or apoptotic are replaced by the replication of neighboring cells, and more significant disruptions to the endothelium (e.g., during injury) are mitigated by circulating endothelial progenitor cells (Mannarino and Pirro, 2008). Atherosclerosis is characterized by cellular processes that promote EC turnover and induce proliferation of the activated endothelium (Xu, 2007). However, whether this proliferation is protective or detrimental has not been fully resolved. The mechanosensitive transcription factor zinc finger and BTB domain containing 46, for instance, inhibits proliferation in quiescent ECs and is downregulated by disturbed flow *in vitro* (Wang et al., 2019a). Hemodynamic signaling also impacts epigenetic pathways, and we have reviewed the impact of epigenetics on atherosclerosis elsewhere (Khyzha et al., 2017). For example, altered flow has been shown to induce DNA methyltransferase 1-mediated hypermethylation of the endothelium and its subsequent inhibition limits atherosclerosis via the cell cycle regulator cyclin A (Zhang et al., 2017). Furthermore, laminar flow-induced autophagy and expression of the deacetylase sirtuin 1 (SIRT1) together inhibit Hippo/YAP signaling to attenuate atherosclerotic plaque formation (Yuan P. et al., 2020), and hypermethylation of eNOS promoter elements under chronic disturbed flow has been shown to contribute to repressed eNOS expression in wildtype mice (Ku et al., 2021).

Flow-sensitive microRNAs have also been identified as key modulators of endothelial proliferation and turnover in atherosclerosis (Kumar et al., 2019), including miR-126-5p, which has been shown to contribute to proliferative reserve in ECs and prevents plaque development through upregulation of Notch signaling (Schober et al., 2014). Curiously, increased expression of miR-126 has been found in atheroprone regions of the endothelium (Zhou et al., 2013). Furthermore, physiological flow conditions have also been observed to promote antiproliferative microRNA activity within the endothelium, whereby KLF2-induced miR-23b represses cyclin H to reduce activity of the cyclin-dependent kinase–activating kinase complex and limit cell cycle progression (Wang et al., 2014). The regulatory interplay of antagonistic, flow-sensitive microRNAs may serve as a mechanism for fine-tuning EC proliferation and warrants further characterization in both quiescent and activated endothelial states.

### Perspectives for the Elucidation of Transcriptional and Epigenetic Regulation in Atherosclerosis

A robust characterization of the transcriptional and epigenetic regulatory elements that contribute to endothelial dysfunction has remained an important challenge for atherosclerosis research and has been recently addressed with next-generation approaches for global EC profiling. Of note, the integration of chromatin immunoprecipitation, chromatin accessibility, and RNA sequencing has enabled identification of diverse DNA regulatory elements associated with disturbed flow and proinflammatory activation (e.g., NF-κB and hypoxia inducible factor 1α, as well as ETS, zinc finger, and activator protein 1 transcription factor families) (Hogan et al., 2017; Bondareva et al., 2019; Alizada et al., 2021). Moreover, combined single-cell RNA sequencing and genome-wide chromatin accessibility assays have been used to profile the genome- and epigenome-wide changes associated with proatherogenic ECs under oscillatory shear stress (Andueza et al., 2020). Likewise, these technologies have allowed for characterization of EC heterogeneity in human atherosclerotic plaque (Depuydt et al., 2020) and identification of coronary artery disease-associated genetic variants in the open chromatin regions of activated ECs (Örd et al., 2021). The future integration of global EC profiling with functional assays will aid in the validation of putative modulators of atherogenesis, which may serve as prospective therapeutic targets for CVD treatment. Notably, real-time monitoring of the vascular endothelium has been enabled with organ-on-a-chip technologies (Sei et al., 2017), allowing for specific and quantifiable testing to elucidate the impact of environmental stimuli on endothelial function. Nevertheless, the complex cellular milieu of the atherosclerotic plaque remains difficult to fully model.

## Alterations in Endothelial Cell Metabolism Contribute to Atherogenesis

Metabolic pathways prominently contribute to EC phenotypes in health and disease. Despite direct exposure to oxygen in the blood, ECs do not utilize oxidative phosphorylation as a primary means of energy production, perhaps because this might enhance oxidative stress and would hamper angiogenesis in hypoxic environments (Eelen et al., 2018). Instead, glycolysis serves as the primary method of energy delivery for ECs, in which 75–85% of ATP is generated via hexokinase 2-mediated phosphorylation of glucose to glucose-6-phosphate and conversion to lactate (Krützfeldt et al., 1990; De Bock et al., 2013; Yu et al., 2017). Fatty acid oxidation, used as a secondary source of energy by ECs, is modulated by carnitine palmitoyltransferase 1A-mediated shuttling of fatty acids to the mitochondria and ATP production via adenosine monophosphate activated protein kinase signaling (Dagher et al., 2001; Currie et al., 2013). Alternatively, fatty acids can be generated within ECs via fatty acid synthase, which has important functions for EC migration, permeability, and eNOS-mediated activity (Wei et al., 2011; Hagberg et al., 2013). Moreover, the proliferative and vasodilatory capacities of the endothelium can also be regulated by the metabolism of amino acids, in which inhibition of glutaminase-mediated conversion of glutamine to α-ketoglutarate represses angiogenesis (Kim B. et al., 2017), and the eNOS-induced conversion of arginine to NO controls vascular tone (Morris Jr, 2009).

Altered EC metabolism has been identified as both a consequence of, and a contributor to, endothelial dysfunction in atherosclerosis. In the quiescent endothelium, laminar shear stress reduces glucose uptake and glycolytic and mitochondrial activity in ECs via KLF2 (Doddaballapur et al., 2015). Conversely, in the diabetic and proatherogenic environment, elevated levels of circulating glucose induce the production of reactive oxygen species, DNA damage, and the accumulation of advanced glycation end products, which contribute to endothelial dysfunction via NF-κB signaling and increased vascular permeability (Theodorou and Boon, 2018). Disturbed flow similarly induces NF-κB and hypoxia inducible factor 1α expression, EC proliferation, and inflammation via upregulation of glycolytic enzymes (Feng et al., 2017), and can also promote EC activation and atherosclerosis through YAP/tafazzin (TAZ) signaling (Wang K.-C. et al., 2016; Wang et al., 2016 L.). Importantly, YAP/TAZ signaling has been shown to induce EC glycolysis, and glycolytic activity can in turn upregulate the YAP/TAZ pathway (Enzo et al., 2015; Bertero et al., 2016; Kim J. et al., 2017), which has been hypothesized to result in a cyclical and sustained pro-inflammatory response in the perturbed endothelium (Theodorou and Boon, 2018). Emerging preclinical studies propose altered EC metabolism as a therapeutic target for mitigating the development of atherosclerosis, including coenzyme Q10-mediated activation of the AMP-activated protein kinase-YAP-optic atrophy protein 1 pathway to promote mitochondrial function and energy metabolism (Xie et al., 2020), and inhibition of the glycolytic regulator 6-phosphofructo-2-kinase/fructose-2,6-biphosphatase 3 to improve plaque stability in atheroprone mice (Poels et al., 2020). Although repressing glycolysis in the activated endothelium remains a promising strategy for treating patients with atherosclerotic CVD, consideration must be given to the complex interactions between genetics and environment (e.g., age, diet, diabetes, and cardiovascular fitness) that shape individual metabolic profiles to allow for patient-tailored therapies.

## Disruption of Endothelial Barrier Promotes Atherosclerosis

In its quiescent state, the vascular endothelium forms a semipermeable barrier between luminal and abluminal environments that allows for selective bidirectional movement of molecules via EC cell-cell junctions, vesicle-mediated transport within ECs, and diffusion between ECs or across endothelial gaps (Cahill and Redmond, 2016). In pioneering studies, LDL and other serum macromolecules were shown to enter the vessel wall using a paracellular route in permeable regions of the vasculature (Weinbaum et al., 1985; Lin et al., 1990). However, more recently, ECs have been implicated in the active transcytosis of LDL via caveolae, scavenger receptor B1, activin receptor-like kinase 1, LDL receptor, and high mobility group box protein 1, which contribute to the proinflammatory accumulation of LDL within the subendothelial space and promote atherogenesis (Zhang X. et al., 2018; Ghaffari et al., 2021). Loss of barrier function also potentiates atherosclerosis by facilitating leukocyte extravasation into the vessel wall through paracellular diapedesis from the vascular lumen, and may promote inflammatory infiltration through the vasa vasorum, contributing to subsequent plaque instability (Mulligan-Kehoe and Simons, 2014; Sluiter et al., 2021). Vascular insults including atherosclerosis, ischemia, and trauma are characterized by the accumulation of pathological proinflammatory mediators, which can induce acute (e.g., due to vascular injury) or chronic disruptions in endothelial permeability (e.g., due to plaque progression). Numerous mechanisms that underlie endothelial barrier disruption have been characterized, including protein kinase C-induced phosphorylation of cell junction proteins, which promotes actin reorganization and increased paracellular flux (Lum and Malik, 1996), and stimulation of myosin light chain kinase by inflammatory factors, which promotes EC retraction via actin-myosin network dynamics (Lum and Malik, 1996). Mediators including histamine, thrombin, and the proinflammatory cytokines interleukin (IL) 1 beta (IL-1β) and tumor necrosis factor α also increase permeability via modulation of tight junctions (e.g., zonula occludens 1 and occludins) and adhesion complexes (Lum and Malik, 1996). Furthermore, prolonged inflammation and oxidative stress can act to disrupt adherens junctions [e.g., vascular endothelial (VE)-cadherin] and gap junctions (e.g., connexins) via decreased NO (Komarova et al., 2017).

Emerging studies continue to uncover novel causal roles for established atherosclerosis mediators in the disruption of endothelial permeability. The mechanosensitive channel Piezo1, previously discussed as a coordinator of vascular structure and activator of integrin α5/NF-κB pathways, also promotes VE-cadherin degradation and increased vascular permeability under altered hemodynamics (Friedrich et al., 2019). Furthermore, the NOD like receptor family pyrin domain containing 3 (NLRP3) inflammasome, discussed in greater detail below as a therapeutic target for inflammation resolution in atherosclerosis, induces a loss of barrier in the diabetic vascular environment (Li X.-X. et al., 2019). Recent findings have also implicated the chemokine C–C motif ligand 8 in promoting atherosclerosis through enhanced permeability via NADPH oxidase 2 and reactive oxygen species (Xue et al., 2021), while the inhibition of insulin-like growth factor-1 signaling has been associated with disrupted endothelial barrier function *in vitro* and elevated atherosclerotic burden in mice (Higashi et al., 2020). The multifaceted roles of these atherogenic factors offer potential advantages for prospective therapies, which may simultaneously target multiple aspects of the atherosclerotic disease processes to limit plaque development (e.g., combined anti-inflammatory and pro-barrier effects of pharmacological agents that inhibit Piezo1 or NLRP3).

## Endothelial Cell Plasticity Contributes to the Atherosclerotic Disease Process

The endothelium is an essential regulator of vascular homeostasis, defined as the balance of vascular injury and repair, and dynamic changes in endothelial phenotype can both potentiate and limit atherogenesis and associated complications (Bäck et al., 2019; Chen P.-Y. et al., 2020). In the setting of chronic inflammatory diseases, including atherosclerosis, ECs undergo complete or partial endothelial-to-mesenchymal transition (EndMT), during which they lose endothelial properties and gain mesenchymal cell characteristics (e.g., extracellular matrix production and contractile function) (Souilhol et al., 2018; Chen P.-Y. et al., 2020). EndMT is driven by proatherogenic stimuli including inflammation and disturbed flow (Chen et al., 2015) and early activation of transforming growth factor beta signaling (Ma et al., 2020), and has garnered interest as an important pathophysiological mechanism for atherosclerotic CVD. Indeed, previous work has linked EndMT to neointimal hyperplasia (Chen et al., 2015; Moonen et al., 2015), increased leukocyte migration (Evrard et al., 2016), lipid uptake by lesional macrophages (Chen et al., 2019), and oxidative stress (Evrard et al., 2016) in the developing plaque (Figure 1). Moreover, statin therapy (Li Y. et al., 2021), histone deacetylase inhibitors (Chen et al., 2021), and microRNA inhibition (Wu et al., 2021) have recently been investigated as potential strategies for limiting EndMT, the latter of which was observed to reduce plaque formation in atherosclerotic mice.

### Perspectives for Future Investigation of Endothelial Plasticity

Future study is warranted to address several outstanding research areas in EndMT and atherosclerosis, including the characterization of EndMT as a discrete versus continuous process, as well as the potential beneficial versus detrimental role of EndMT in plaque vulnerability. First, the proatherogenic endothelium has recently been shown to exist in a “metastable or partial” state of EndMT, where perturbed ECs perform both endothelial and mesenchymal functions (Helmke et al., 2019; Fledderus et al., 2021). The metastable nature of EndMT may be further elucidated with next-generation technologies that profile the atherosclerotic plaque and neighboring regions at the single-cell level, as recently used in studies of disturbed flow and endothelial reprogramming (Andueza et al., 2020) and diabetic atherogenesis (Zhao et al., 2021), as well as through the incorporation of computational models that predict endothelial activation and EndMT in various genetic and environmental conditions (Weinstein et al., 2020). Second, although augmentation of EndMT has previously been associated with increased plaque progression (Chen et al., 2015), emerging studies have highlighted a potential protective role of EndMT by maintaining plaque stability in the absence of SMC-derived myofibroblast-like cells (Evrard et al., 2016; Newman et al., 2021). Evidently, a more nuanced understanding of EndMT in the context of plaque development and rupture is required before therapeutic inhibition of EndMT is considered for the treatment of atherosclerotic CVD.

## Endothelial Cells Are Key Players in Vascular Cell-Cell Communication During Atherosclerosis

Vascular cell populations including ECs, monocytes, macrophages, and SMCs have well-established roles in driving atherosclerosis. ECs orchestrate the cellular interactions between these cell populations through the local expression of adhesion proteins and secretion of signaling molecules (Raines and Ferri, 2005; Liebner et al., 2006; Okamoto and Suzuki, 2017). The intercellular processes that mediate this pathophysiology have been a focus of previous studies, which have characterized EC-EC and EC-leukocyte communication via adherens junctions (e.g., VE-cadherin), tight junctions (e.g., junctional adhesion molecules), and other adhesion proteins including occludin, claudins, and platelet endothelial cell adhesion molecule (Liebner et al., 2006; Reglero-Real et al., 2016). Furthermore, a diverse group of EC-derived cytokines, chemokines, and other molecules (e.g., NO and endothelin-1) regulate vascular function by controlling proatherogenic processes such as leukocyte activation and SMC proliferation (Raines and Ferri, 2005; Gimbrone and García-Cardeña, 2016; Fledderus et al., 2021). Inflammatory cell recruitment to the activated endothelium is coordinated by the expression of EC adhesion proteins such as E-selectin, VCAM-1, and intercellular adhesion molecule-1 (ICAM-1), which facilitate leukocyte rolling and extravasation into the intimal layer, as well as the chemotactic factor monocyte chemoattractant protein-1, which attracts circulating monocytes, and the mitogen macrophage colony-stimulating factor, which stimulates monocyte proliferation and differentiation to intimal macrophages (Kleemann et al., 2008; Fledderus et al., 2021). Additionally, the proinflammatory polarization of macrophages has been associated with EndMT (Wu et al., 2017) and sprouting angiogenesis, the latter of which supports plaque progression through increased supply of oxygen and nutrients (Camaré et al., 2017; Graney et al., 2020). The developing atherosclerotic lesion is further characterized by intimal hyperplasia and neointimal formation, which are mediated by decreased production of EC-derived NO and a resultant increase in SMC proliferation, in conjunction with EndMT (Fledderus et al., 2021).

### Extracellular Vesicles Mediate Paracrine Communication Between Endothelial Cells in Atheroprone and Atheroprotective Environments

In recent years, vascular cell-cell communication mediated by the secretion of extracellular vesicles (i.e., EVs; nano-sized packages of proteins, mRNAs, noncoding RNAs, and lipids) has emerged as an important research area for the pathogenesis and treatment of atherosclerosis (Charla et al., 2020; Chen Y.-T. et al., 2020; Wang H. et al., 2020). Three broad categories of EVs – exosomes (30–150 nm in diameter), microparticles (100 nm–1 μm in diameter), and apoptotic bodies (1–5 μm in diameter) – have been shown to modulate vascular function in atherogenesis via intercellular signaling among ECs, as well as between ECs and other immune and non-immune cell types (He et al., 2018; Li M. et al., 2018; Charla et al., 2020; Peng et al., 2020) (Figure 1). Within the vascular endothelium, EC-derived EVs with distinct microRNA content have been observed to both induce (Jansen et al., 2013; Arderiu et al., 2015) and inhibit angiogenesis (Ou et al., 2011; Liang et al., 2017, 2) via paracrine regulation of nearby ECs, whereby EVs fuse to recipient cells, and the release of EV-derived microRNAs allows for silencing of complementary mRNA targets. Moreover, the uptake of EC-secreted microparticles by neighboring ECs was shown to promote ICAM-1 expression and monocyte adhesion via miR-222 in diabetic conditions (Jansen et al., 2015), and EV-derived apoptotic bodies containing miR-126 were found to reduce macrophage content in plaque and limit atherosclerotic burden (Zernecke et al., 2009). The specific role of the proatherogenic environment in governing EV loading and function remains of interest for future study, and characterization of this environment has begun with exposure of donor ECs to proatherogenic stimuli (i.e., oxLDL and IL-6), which were observed to decrease thrombospondin 1 and increase angiogenesis in recipient ECs via EV-derived miR-92a-3p (Liu Y. et al., 2019).

### Perturbed Intercellular Communication Between Endothelial Cells and Immune and Non-Immune Cells Potentiates Atherogenesis

Beyond the endothelium, EC-EVs regulate both immune and non-immune vascular cell populations involved in the development of atherosclerosis (Li M. et al., 2018; He et al., 2018) and circulating EVs may have systemic effects at distant sites (Bär et al., 2019; Liu et al., 2021). Exosomal microRNAs mediate crosstalk between ECs and macrophages by suppressing (e.g., miR-10a) (Njock et al., 2015) or promoting monocyte activation (e.g., miR-155) (He et al., 2018), reducing macrophage infiltration, and delaying plaque progression (e.g., miR-126, miR-210, and miR-216) (Wang et al., 2019b). In turn, monocyte- and macrophage-derived EVs have been shown to induce EC apoptosis and promote murine atherosclerosis through elevated blood lipids, oxidative stress, and inflammation (Aharon et al., 2008; Li K. et al., 2021), as well as by reducing proliferation and angiogenesis in human coronary artery ECs via miR-503-5p (Wang et al., 2021). Reciprocal communication between cells of the vasculature has been similarly observed in ECs and SMCs, where EC-secreted EVs induce both atheroprone (Boyer et al., 2020; Yuan X. et al., 2020; Zhang Z. et al., 2020) and atheroprotective SMC phenotypes (Hergenreider et al., 2012; Xiang et al., 2021), while SMC-derived EVs govern endothelial migration under stimulation by platelet-derived growth factor (Heo et al., 2020) and can increase endothelial permeability and potentiate atherosclerosis via miR-155 (Zheng et al., 2017).

### Perspectives for the Characterization of Circulating Extracellular Vesicles

EC-EVs that are secreted into systemic circulation may serve as biomarkers for CVD and have the potential to govern cell behaviour at distant areas of the vasculature, although definitive evidence for this regulation is lacking (Bär et al., 2019; Liu et al., 2021). Preliminary animal studies have demonstrated that microRNAs secreted by circulating blood cells, and presumably contained within EVs, can modulate SMC activity in atherogenesis (Shan et al., 2015) and have proapoptotic and antiproliferative effects on ECs (Chu et al., 2017). Further investigation is warranted to determine whether circulating EVs are present in sufficient quantities to elicit systemic effects in patients and if their regulatory functions extend to other CVDs (e.g., cardiac fibrosis and ischemic heart disease), as well as to identify the precise mechanisms by which circulating EVs promote vascular dysfunction in recipient cells. Notably, future study is also required to determine whether EVs undergo transcytosis or pass through intercellular or intracellular gaps to the subendothelial space and elicit direct effects on medial cells, or are endocytosed by ECs, which then serve as indirect mediators of altered vascular function. The continued integration of EV profiling in patients with atherosclerosis (i.e., local EVs in plaque; circulating EVs in plasma) with experimental models of EV activity will shed light on the causal role of EVs and EV-contents in the setting of atherosclerotic CVD. Such models will require the transition from *in vitro* co-culture towards *in vivo* tracking approaches to allow for a robust, physiological characterization of EV activity, which will be made possible with advances in the visualization of EV release and uptake (Durak-Kozica et al., 2018; Oesterreicher et al., 2020). With respect to the diagnostic potential of vascular EVs, the application of machine learning strategies to predict CVD from circulating EV biomarkers has garnered recent interest (Burrello et al., 2020; Castellani et al., 2020) and will likely play an important role in the development of precision cardiovascular medicine over the coming decades.

## The Vascular Endothelium as an Emerging Therapeutic Target for Atherosclerosis

Present therapeutic strategies to limit atherosclerosis and plaque rupture are broadly categorized as those which reduce atherosclerotic risk (e.g., lipid-lowering and antihypertensive agents) and those which prevent associated complications (e.g., antithrombotic agents) (Fledderus et al., 2021). Lipid-lowering therapies, including 3-hydroxy-3-methyl-glutaryl-coenzyme A reductase inhibitors (i.e., statins) and proprotein convertase subtilisin/kexin type 9 inhibitors, reduce circulating levels of LDL-cholesterol and thus oxLDL in the vessel wall, and decrease the incidence of severe events in CVD (Silverman et al., 2016; Mach et al., 2020), although these agents are not sufficient to prevent plaque formation (Tran-Dinh et al., 2013; Moss and Ramji, 2016). Moreover, a spectrum of anti-atherogenic effects of statins on the endothelium has been previously discussed in detail (Xu et al., 2021), and includes reduced inflammation through NF-κB blockade (Greenwood and Mason, 2007), inhibition of EC apoptosis via Janus kinase 2/signal transducer and activator of transcription 3 signaling (Wang K. et al., 2020), protection against EndMT via Kruppel-like factor 4/miR-483 (He et al., 2017), epigenetic modulation of ECs through histone modification (Mohammadzadeh et al., 2020), and increased NO production via hydrogen sulfide and eNOS (Citi et al., 2021; Xu et al., 2021). Notably, the statin-mediated mechanisms that contribute to elevated eNOS activity include increased eNOS transcription via KLF2 (Parmar et al., 2005), improved eNOS mRNA stability via polyadenylation (Kosmidou et al., 2007), and increased eNOS phosphorylation via phosphatidylinositol 3-kinase/Akt signaling (Kureishi et al., 2000). Other anti-atherogenic treatments have also been observed to mitigate endothelial dysfunction by increasing NO production, such as antihypertensive agents (e.g., angiotensin-converting enzyme inhibitors and angiotensin II receptor blockers) (Silva et al., 2019), antihyperglycemic drugs (e.g., insulin) (Muniyappa et al., 2008), and antioxidants (e.g., streptozotocin) (Varadharaj et al., 2017). In experimental settings, treatments for atherosclerosis have largely focused on antagonizing broad inflammatory pathways (e.g., IL-1β and 5-lipoxygenase) (Tardif et al., 2010; Ridker et al., 2017). Therapeutic agents that specifically target the perturbed endothelium to resolve inflammation and limit atherosclerosis, however, have not yet been fully realized in clinical studies (Fledderus et al., 2021).

### Promoting Inflammation Resolution to Mitigate Atherosclerosis

The NLRP3 inflammasome is now recognized as a key player in the coordination of vascular inflammation and onset of atherogenesis (Duewell et al., 2010; Jin and Fu, 2019). NLRP3-mediated induction of atherosclerosis has been associated with a host of causal factors including hypoxia (Folco et al., 2014), cholesterol crystals and oxLDL (Duewell et al., 2010), and disturbed flow (Xiao et al., 2013). Activation of NLRP3 subsequently leads to the maturation of proinflammatory cytokines (e.g., IL-1β and IL-18), increases the migration and lipid loading of macrophages (Li X. et al., 2014), and promotes pyroptosis, a proinflammatory form of programmed cell death that contributes to the release of additional inflammatory mediators (Bergsbaken et al., 2009). Therapeutic agents that directly inhibit the NLRP3 inflammasome, including the natural compound arglabin (Abderrazak et al., 2015), colchicine (Fernando et al., 2017), and the small molecule inhibitor MCC950 (van der Heijden et al., 2017), have been shown to ameliorate endothelial inflammation and atherosclerosis in preclinical studies. Moreover, adjunct colchicine therapy has entered clinical trials for repressing vascular inflammation in acute coronary syndrome (Bouabdallaoui et al., 2020) and coronary artery disease (Nidorf et al., 2013; Kajikawa et al., 2019; Hays et al., 2021). Previous trials have employed several outcomes related to improved cardiovascular health, including CVD endpoints, serum inflammatory markers, and proxy measures for endothelial dysfunction including flow-mediated vasodilation and exercise-induced coronary blood flow. Although the latter two of these measures remain the gold standard for clinical assessment of the perturbed endothelium (Flammer et al., 2012; Xu et al., 2021), they rely on endothelial responsiveness to vasodilatory or vasoconstrictive manipulation and do not adequately capture changes in EC phenotype during atherogenesis (e.g., permeability, metabolism, EndMT, or intercellular communication). Emerging studies have begun to address this limitation by applying machine learning techniques to predict CVD events from clinical data, CT imaging, and circulating biomarkers, including matrix metalloproteinase 9 and polymeric immunoglobulin receptor (Tamarappoo et al., 2021), very low-density lipoprotein and leucine (Coelewij et al., 2021), and serum EVs (Burrello et al., 2020; Castellani et al., 2020). Alongside functional assessment of the endothelium, robust models for CVD prediction have the potential to identify subclinical disease (Coelewij et al., 2021), aid in risk stratification, and inform treatment and prevention strategies.

Recent studies have also highlighted the potential of endogenous specialized proresolving mediators (SPMs) as anti-inflammatory therapies for limiting endothelial inflammation in CVD (Fredman and Tabas, 2017; Xu et al., 2021). Resolvin D1 (RvD1) belongs to a family of SPMs formed from metabolic processing of polyunsaturated docosahexaenoic acid and eicosapentaenoic acid, and is synthesized by ECs under proinflammatory conditions including stimulation with oxLDL (Dufour et al., 2018). Treatment of ECs with RvD1 has been observed to repress lipopolysaccharide- and cholesterol crystal-stimulated EC-monocyte interactions *in vitro* by downregulating NFκB-ICAM-1/VCAM-1 signaling (Chattopadhyay et al., 2018; Pichavaram et al., 2019), and attenuates leukocyte trafficking to the endothelium in mice fed a cholesterol rich diet (Pichavaram et al., 2019). Beyond the resolvin family, the SPMs maresin 1 and lipoxin A4 have likewise been observed to disrupt leukocyte adhesion to activated ECs (Filep et al., 1999; Chatterjee et al., 2014), suggesting that endogenous production or exogenous administration of diverse SPM metabolites may have therapeutic benefits for limiting endothelial activation in the setting of atherosclerosis.

Notably, the potential therapeutic impact of promoting inflammation resolution through efferocytosis (i.e., the clearing of apoptotic cells) is being established with preclinical studies, wherein atherosclerotic plaque progression has been attenuated by proefferocytic cluster of differentiation 47 (CD47)-blocking therapies (Kojima et al., 2016; Flores et al., 2020) or activation of the macrophage cell surface receptor MER proto-oncogene, tyrosine kinase (Thorp et al., 2008; Cai et al., 2018). While these effects appear to be mediated by lesional macrophages, ECs could facilitate the transport of inflammation resolving agents to cells within the developing plaque. Indeed, a proefferocytic and anti-atherosclerotic nanoparticle therapy has been previously hypothesized to function by uptake within circulating monocytes or delivery to macrophages within the intima (Flores et al., 2020), the latter of which would require the monocyte-independent transport of nanoparticles across the endothelium. In this role, ECs may contribute to therapeutic delivery by modulating vascular permeability and trafficking nanoparticles to macrophages via EC-secreted EVs. Clinically, the promise of inflammation resolving strategies has been highlighted in a subset of patients enrolled in a phase 1b-2 trial of the proefferocytic anti-CD47 antibody magrolimab for treating B-cell non-Hodgkin’s lymphoma, where vascular inflammation was significantly reduced in the carotid arteries of patients with CVD after 9 weeks of treatment (Jarr et al., 2021).

### Epigenetic Interventions to Limit Atherosclerotic Plaque Progression

**FIGURE 2:**
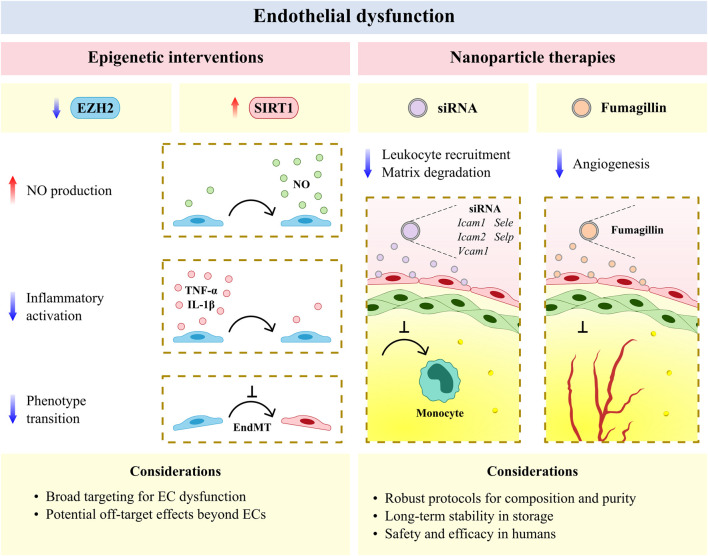
The vascular endothelium is an emerging therapeutic target for atherosclerotic cardiovascular disease. Epigenic interventions including enhancer of zeste homologue 2 (EZH2) antagonism and sirtuin 1 (SIRT1) agonism allow for broad targeting of endothelial cell (EC) dysfunction but may have undesirable effects in other cell types and tissues. Anti-inflammatory and anti-angiogenic nanotherapies have reduced off-target effects and increased efficacy per dose, but require rigorous protocols for assessing composition and purity, as well as the development of long-term stability in storage and safety and efficacy in humans with cardiovascular disease. EndMT, endothelial-to-mesenchymal transition; Icam1, intercellular adhesion molecule 1; Icam2, intercellular adhesion molecule 2; IL-1β, interleukin 1 beta; NO, nitric oxide; Sele, selectin E; Selp, selectin P; siRNA, small interfering RNA; TNF-α, tumor necrosis factor alpha; Vcam1, vascular cell adhesion molecule 1.

Epigenetic interventions allow for broad targeting of the diverse mechanisms underlying endothelial dysfunction and have shown recent promise as anti-atherosclerotic therapies (Fledderus et al., 2021). The methyltransferase enhancer of zeste homologue 2 (EZH2) and histone deacetylase SIRT1 respectively induce and limit endothelial dysfunction in murine models of CVD (Zhou et al., 2011; Lv et al., 2016) and represent novel avenues for epigenetic treatment. EZH2 antagonism and SIRT1 agonism similarly increase NO production (Gracia-Sancho et al., 2010; Kumar et al., 2013) and decrease EC activation (Zhou et al., 2011; Wang J. et al., 2020) and EndMT (Maleszewska et al., 2016; Liu Z.-H. et al., 2019) in experimental models of CVD (Figure 2). More recently, natural antioxidants including epigallocatechin and naringenin have been shown to reduce oxidative damage in ECs via SIRT1 (Li H. et al., 2021; Pai et al., 2021), and an intermittent fasting regimen was found to ameliorate vascular dysfunction in a murine model of diabetes by activating the SIRT1 pathway (Hammer et al., 2021). Small molecule agents that target EZH2 and SIRT1 are currently available or under clinical study in several disease areas (e.g., oncology, dermatology, and nephrology) (Ganesan et al., 2019; Fledderus et al., 2021), and may be investigated as potential anti-atherosclerotic agents by incorporating CVD endpoints into ongoing clinical trials. Beyond their desired therapeutic potential, the unintended effects of genome-wide modification via epigenetic agents also warrant consideration. Future studies may improve upon the specificity of these agents by making use of targeted CRISPR/Cas9 epigenome-editing techniques for epigenetic modifications associated with CVD risk loci (Cano-Rodriguez et al., 2016; Cano-Rodriguez and Rots, 2016; Xu et al., 2018). Alternatively, targeted EC therapies, as discussed below, may be considered for the resolution of vascular inflammation at the site of the activated endothelium in atherosclerosis. Prospective agents for targeted resolution include ICAM-1- or VCAM-1-targeted nanocarriers, which have previously been employed to limit neurovascular (Lutton et al., 2017; Marcos-Contreras et al., 2020) and pulmonary inflammation (Li S. et al., 2018; Park et al., 2021) in experimental models of disease.

### Nanoparticle Therapies for the Targeted Resolution of Endothelial Dysfunction in Atherosclerosis

Advances in the development of nanoparticles (on a scale of <0.1 μm) have likewise afforded new opportunities for anti-atherosclerotic therapies that resolve endothelial dysfunction (Flores et al., 2019; Marchio et al., 2019; Chen L. et al., 2020). Vascular nanotherapies have been broadly classified as those which resolve inflammation and dysfunction in efferocytosis, limit plaque neovascularization and neointimal growth, modulate lipid metabolism, and decrease thrombosis (Flores et al., 2019). Several of these therapies have been shown to target the perturbed endothelium in experimental models of CVD, including the encapsulation of five cell adhesion molecule small interfering RNA, which reduced leukocyte migration to plaque and suppressed post-myocardial infarction inflammation in murine models (Sager et al., 2016). Integrin-targeted nanoparticles containing the antiangiogenic compound fumagillin have likewise been shown to limit neovascularization in mice and demonstrate prolonged activity when combined with statin treatment (Winter et al., 2006; Winter et al., 2008) (Figure 2). More recently, engineered endothelial adrenoreceptor- (Ul Ain et al., 2017), VCAM-1- (Distasio et al., 2021), and P-selectin-targeted nanoparticles (Mocanu et al., 2021), which respectively carried the genes eNOS and IL-10 and receptor for advanced glycation end products-silencing RNA, have been found to localize to ECs and resolve inflammation within the murine atherosclerotic plaque. Nano-selenium particles have also been observed to improve endothelial dysfunction in murine atherosclerosis via Na^+^/H^+^ exchanger 1 inhibition (Zhu et al., 2019). Despite the therapeutic advantages offered by nanoparticles (e.g., reduced off-target effects and increased efficacy per dose), several methodological and biological challenges remain for clinical translation (Flores et al., 2019). Importantly, robust protocols are required to determine the composition and purity of nanoparticles derived using various formulations (e.g., lipid or polymeric). Furthermore, the long-term stability of nanoparticles, as well as their safety and selectivity for the activated endothelium in patients with atherosclerosis, must be assessed before these agents are clinically adopted. Nevertheless, vascular nanotherapies remain a promising avenue for the targeted resolution of endothelial dysfunction and reduction of plaque progression in atherosclerotic CVD, with encouraging evidence being generated for the systemic administration of nanoparticle-enveloped small interfering RNA in nonhuman primates to inhibit EC gene expression in multiple organs (Khan et al., 2018).

## Conclusion and Future Perspectives

An emerging body of literature has implicated the vascular endothelium as an essential driver of atherosclerosis, a disease process that contributes to substantial mortality and healthcare burden among aging populations. These discoveries shed light on diverse cellular mechanisms that underlie endothelial activation, dysfunctional cell-cell communication, and perturbed vascular homeostasis in atherogenesis. Moreover, they highlight novel therapeutic targets and delivery methods with significant potential to prevent or limit plaque development in patients with atherosclerotic CVD. Although a broad spectrum of intra- and intercellular processes has been implicated in preclinical models of endothelial dysfunction and atherosclerosis, several methodological challenges remain to effectively translate this research to clinical practice. First, the generalizability of findings between diverse *in vitro* and *in vivo* models of endothelial activation and atherosclerosis remains an important consideration to identify strategies that hold significant therapeutic potential for distinct plaque locations and diverse patient groups. Additionally, the safety, efficacy, timing, and sustained response of candidate treatments must be considered, as the atherosclerotic disease process develops over decades and necessitates long-term intervention. A robust integration of experimental techniques (e.g., high-throughput EC profiling with functional validation), data sources (e.g., human samples with experimental models), and cutting-edge computational methods (e.g., machine learning) will be required for the creation of next-generation biomarkers and therapies that effectively mitigate atherosclerotic CVD in vulnerable patients.

## Abbreviations

CD47, cluster of differentiation 47; CVD, cardiovascular disease; EC, endothelial cell; EndMT, endothelial-to-mesenchymal transition; eNOS, endothelial nitric oxide synthase; EV, extracellular vesicle; EZH2, enhancer of zeste homologue 2; ICAM-1, intercellular adhesion molecule-1; IL, interleukin; IL-1β, interleukin 1 beta; KLF2, Kruppel-like factor 2; LDL, low-density lipoprotein; NF-κB, nuclear factor kappa B; NLRP3, NOD like receptor family pyrin domain containing 3; oxLDL, oxidized low-density lipoprotein; RvD1, resolvin D1; SIRT1, sirtuin 1; SMC, smooth muscle cell; SPM, specialized proresolving mediator; TAZ, tafazzin; VCAM-1, vascular cell adhesion molecule 1; VE-cadherin, vascular endothelial-cadherin; YAP, yes-associated protein.
